# Immediate and Persistent Transcriptional Correlates of Long-Term Sensitization Training at Different CNS Loci in *Aplysia californica*

**DOI:** 10.1371/journal.pone.0114481

**Published:** 2014-12-08

**Authors:** Samantha Herdegen, Catherine Conte, Saman Kamal, Robert J. Calin-Jageman, Irina E. Calin-Jageman

**Affiliations:** Neuroscience Program, Dominican University, River Forest, Illinois, United States of America; Universitaet Regensburg, Germany

## Abstract

Repeated noxious stimulation produces long-term sensitization of defensive withdrawal reflexes in *Aplysia californica*, a form of long-term memory that requires changes in both transcription and translation. Previous work has identified 10 transcripts which are rapidly up-regulated after long-term sensitization training in the pleural ganglia. Here we use quantitative PCR to begin examining how these transcriptional changes are expressed in different CNS loci related to defensive withdrawal reflexes at 1 and 24 hours after long-term sensitization training. Specifically, we sample from a) the sensory wedge of the pleural ganglia, which exclusively contains the VC nociceptor cell bodies that help mediate input to defensive withdrawal circuits, b) the remaining pleural ganglia, which contain withdrawal interneurons, and c) the pedal ganglia, which contain many motor neurons. Results from the VC cluster show different temporal patterns of regulation: 1) rapid but transient up-regulation of *Aplysia* homologs of C/EBP, C/EBPγ, and CREB1, 2) delayed but sustained up-regulation of BiP, Tolloid/BMP-1, and sensorin, 3) rapid and sustained up-regulation of Egr, GlyT2, VPS36, and an uncharacterized protein (LOC101862095), and 4) an unexpected lack of regulation of *Aplysia* homologs of calmodulin (CaM) and reductase-related protein (RRP). Changes in the remaining pleural ganglia mirror those found in the VC cluster at 1 hour but with an attenuated level of regulation. Because these samples had almost no expression of the VC-specific transcript sensorin, our data suggests that sensitization training likely induces transcriptional changes in either defensive withdrawal interneurons or neurons unrelated to defensive withdrawal. In the pedal ganglia, we observed only a rapid but transient increase in Egr expression, indicating that long-term sensitization training is likely to induce transcriptional changes in motor neurons but raising the possibility of different transcriptional endpoints in this cell type.

## Introduction

How are long-term memories maintained despite molecular turnover in the central nervous system? The answer to this question seems to depend, in part, on learning-induced changes in gene expression. In a wide range of species and learning paradigms, training that produces long-term memory also evokes changes in neuronal gene expression [1]–[3]. Moreover, blocking changes in gene expression has been repeatedly shown to impair the formation of long-term memory [4]–[8]. Thus, there is considerable interest in elucidating the specific transcriptional changes that accompany the encoding and maintenance of long-term memory.

**Figure 1:**
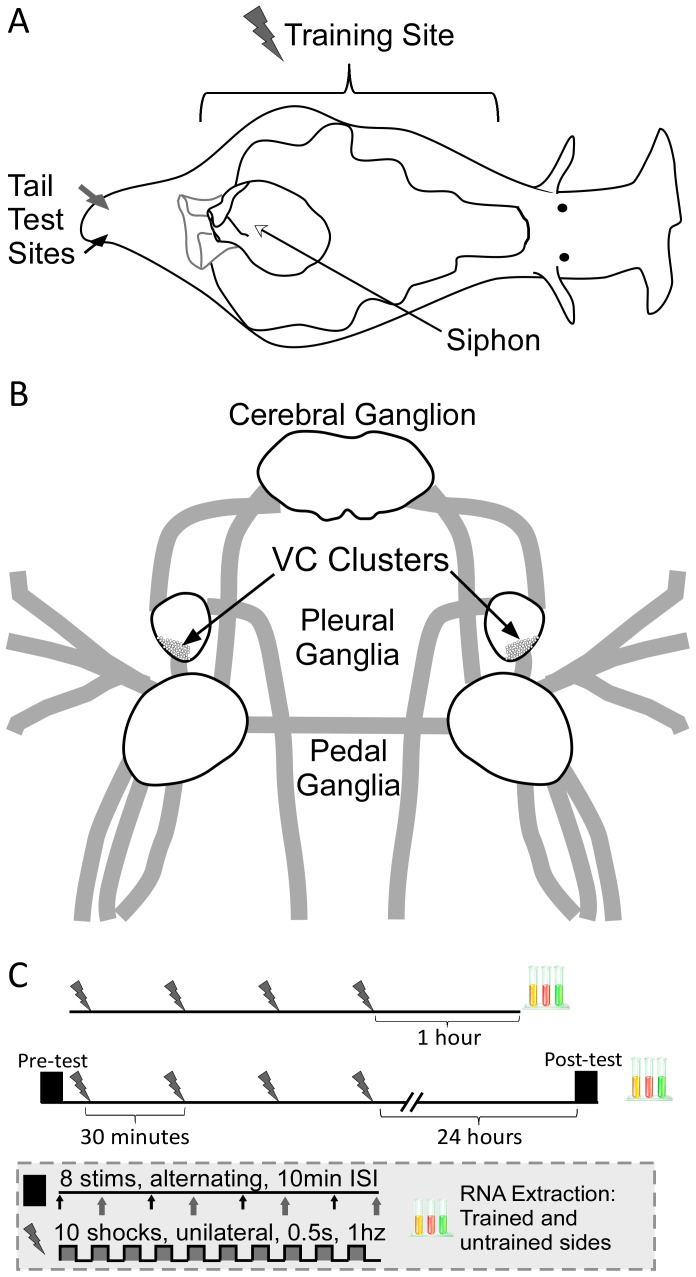
Long-term sensitization overview. A) Long-term sensitization is evoked in *Aplysia* by applying a strong shock (90mA) over most of one side of the body (training site). This produces a robust increase in the duration of defensive withdrawals evoked by innocuous stimulation to both the training site (site-specific sensitization) and to untrained sites on the same side of the body (generalized sensitization). In this paper, generalized sensitization was measured via the tail-elicited siphon withdrawal reflex (T-SWR). The T-SWR reflex is evoked by innocuous electrical shock (2 mA) to the left or right tail (tail test sites). This produces a defensive withdrawal of the siphon (grey) which is measured as the duration of contraction. B) CNS components related to defensive withdrawal reflexes. Defensive withdrawal reflexes are mediated by a number of cell types including: 1) VC nociceptors, which are located in the VC cluster within the pleural ganglia, 2) excitatory and inhibitory interneurons in the pleural ganglia, 3) motor neurons in the pedal ganglia, and 4) siphon, gill, and mantle motor neurons in the abdominal ganglia (not shown). C) Experimental protocol. Animals were given long-term sensitization training consisting of four rounds of noxious shock applied to one side of the body at 30 min intervals. CNS samples were then harvested either 1 hour or 24 hours after training. For animals harvested 24 hours after training, T-SWR behavior was also measured before and 24 hours after training.

Sensitization in the marine mollusk *Aplysia californica* has proven a fruitful paradigm for studying the transcriptional mechanisms of long-term memory (Fig. 1A). Sensitization is an increase in reflex responsiveness due to noxious stimulation [9]. This non-associative form of memory is observed across the entire animal kingdom [10]. Mechanistically, sensitization is thought to reflect the operation of basal plasticity mechanisms from which more complex forms of learning have evolved [11]. Moreover, sensitization in *Aplysia* shares many behavioral, physiological, and molecular characteristics with aspects of chronic pain in humans and other mammals [12], [13], and research in *Aplysia* has proven informative for helping to guide research into this important clinical problem [14].

A particular advantage of studying sensitization in *Aplysia* is that it can be induced and expressed unilaterally, by applying noxious shock to one side of the body (Fig. 1A). This produces a unilateral increase in the duration of defensive reflexes in *Aplysia* [15], [16] which can include changes in the siphon-withdrawal reflex (SWR) and the tail-withdrawal reflex (TWR). With repeated exposure to noxious stimulation, a long-term sensitization memory is induced on the trained side of the body which lasts more than 24 hours and requires changes in transcription [17]. Unilateral sensitization training allows powerful within-subjects designs in which these transcriptional changes can be compared from the trained and untrained side of the same animal [18].

Although long-term sensitization training is simple, it produces complex changes in behavior. At the site of training, there is site-specific sensitization which can be observed as a change in the duration of SWRs elicited from the same location that was trained [16], [19]. In addition, sensitization generalizes beyond the region of training. For example, generalized sensitization can be observed in SWRs evoked by tail stimulation even when the tail is not included as a training site [20]. Finally, long-term sensitization training produces additional long-lasting changes in behavior, including a persistent increase in heart rate [21] and a persistent decrease in feeding behavior [22].

The mechanisms mediating the behavioral effects of long-term sensitization training can be readily studied in *Aplysia* because the neural circuitry underlying defensive withdrawal of both the siphon and the tail (Fig. 1B) are relatively well defined [23]. Sensory input is mediated by the VC nociceptors in the pleural ganglia [24] as well as a set of low-threshold mechanoreceptors whose cell bodies have not yet been located [25]–[28]. VC sensory activity is then relayed both directly to tail motor neurons in the pedal ganglia [24] and indirectly through an excitatory interneuron [29] that also activates siphon motor neurons [30] in the abdominal ganglion. Inhibitory interneurons also provide lateral inhibition across the VC nociceptors and inhibit tail motor neurons [31]–[33].

During long-term sensitization training, the noxious stimulus produces robust activation of the VC nociceptors that innervate the training site as well as prolonged depolarization of motor neurons mediating defensive withdrawal [19]. In addition, sensitization training activates an interconnected network of serotonergic neurons [34], producing diffuse serotonin release throughout much of the CNS. Particular targets of serotonin release include the cell bodies of the VC nociceptors in the pleural ganglia and the synaptic contacts of these neurons with motor neurons in the pedal ganglia [35].

The physiological responses during sensitization training lead to long-lasting physiological changes at multiple loci related to defensive withdrawal. Tail motor neurons exhibit a persistent decrease in resting membrane potential coupled with increased excitability [36]. VC nociceptors are also strongly regulated, exhibiting increased excitability [15], [19], [36], spike narrowing [37], and long-term facilitation of their synaptic contacts [19], [36]–[38]. The mechanisms underlying VC plasticity seems to depend in part on their relationship with the site of training. For VC neurons with receptive fields outside the training site, no activity is induced during training and thus the physiological effects are presumably due to serotonergic modulation alone. For VC neurons with receptive fields within the site of training, serotonergic modulation is paired with strong activation, and these combined events produces stronger physiological changes [16] that are at least somewhat distinct mechanistically from those induced by serotonin alone [39].

While the level of VC activity is an important factor in determining the plasticity induced by sensitization training, mere exposure to repeated pulses of serotonin produces heterosynaptic long-term facilitation (LTF) of VC synapses in both isolated ganglia and cell culture [40], [41], mimicking the effects of long-term sensitization training. LTF has thus provided an *in vitro* model for studying the mechanisms of processes related to generalized sensitization. Work with this model has shown that LTF is induced by signal-transduction cascades that converge on the activation of CRE-response elements through the recruitment of the transcription factor ApCREB1 and de-activation of the transcriptional repressor ApCREB2 [42]. This then leads to multiple waves of transcriptional change. To date, over 15 specific transcriptional changes have been found to occur at different time points after the long-term sensitization training or serotonin exposure.

Although the physiological changes that follow long-term sensitization training are known to be distributed across multiple cell types related to defensive withdrawal, little is known about how the transcriptional correlates of sensitization are distributed in these circuits. Some changes have been specifically characterized in the VC nociceptors of the pleural ganglia either through cell culture [43] or by microdissecting out the “sensory wedge” [18], the distinctive, wedge-shaped cluster of VC cell bodies which forms a somatotopic map of the *Aplysia* body [24], [44]. Most transcriptional analyses after sensitization training, however, have been conducted on whole pleural ganglia [45], which could reflect transcriptional changes in the VC nociceptors, T-SWR interneurons, other neurons with defensive functions (e.g., the giant neuron LPl1 in the left pleural ganglion, which stimulates defensive mucus secretion [46]), or even changes in neurons unrelated to defensive withdrawal. Moreover, there do not seem to have been any previous attempts to determine if motor neurons undergo transcriptional regulation in response to long-term sensitization training.

Here we begin an investigation into how the transcriptional changes following sensitization training are expressed in different CNS loci related to defensive withdrawal. Specifically, we collected samples 1 or 24 hours after long-term sensitization training (Fig. 1C) from three different locations: 1) the VC cluster of the pleural ganglia, which represents VC nociceptors cell bodies exclusively, 2) the remaining pleural ganglia (referred to hereafter as rPl), which contains T-SWR interneurons [29], [32], [47], processes of the VC neurons, and other neurons potentially unrelated to defensive withdrawal [46], [48], [49], and 3) the pedal ganglia, which contains motor neurons innervating the tail [24] and much of the rest of the body [50], processes of the VC neurons, and other uncharacterized neuron types. Although only the VC cluster provides a completely homogenous sample of sensory neurons known to mediate defensive withdrawal reflexes, the other tissues provide a starting point for identifying transcriptional changes which might be specific to interneurons or motor neurons that participate in defensive reflexes.

We surveyed all 10 transcripts which have been previously identified as rapidly regulated following long-term sensitization training [18], [51]–[57] as well as two transcripts known to exhibit delayed up-regulation after training (Table 1). For several of these transcripts, our data also provide the first test for VC-specific and/or long-term regulation following sensitization training.

**Table 1:** List of transcripts studied and summary of previous and current results.

			Regulation after Sensitization Training					
			Prior Results			Current Results		
Transcript	Short Name	Accession	Early/Late			Early/Late		
BiP/GRP78 [43]	ApBiP	NM_001204652	none	/	↑	none	/	↑
CCAAT/enhancer binding protein [44]	ApC/EBP	NM_001204463	↑	/	none	↑	/	none
CCAAT/enhancer-binding protein gamma-like [45]	ApC/EBPγ	XM_005105406	↑	/	??	↑	/	none
Calmodulin [12]	ApCaM	NM_001204580	↑	/	??	none	/	none
cAMP responsive element-binding protein [46]	ApCREB1	NM_001256437	↑	/	none	↑	/	none
Early growth response protein 1-like [45]	ApEgr	NM_001281796	↑	/	↑	↑	/	↑
Sodium- and chloride-dependent glycine transporter 2-like [45]	ApGlyT2	XM_005092349	↑	/	??	↑	/	↑
Uncharacterized LOC101862095 [45]	Loc101862095	XM_005113453	↑	/	??	↑	/	↑
Reductase-related protein [12]	ApRRP	NM_001204605	↑	/	??	none	/	none
Sensorin* [47]	sensorin	NM_001204654	??	/	??	none	/	↑
Tolloid/BMP-1 [48]	ApTBL-1	NM_001204563	↑	/	??	none	/	↑
Vacuolar protein-sorting-associated protein 36-like [45]	ApVPS36	XM_005112280	↑	/	??	↑	/	↑

Transcripts are annotated with reference that first identified regulation by long-term sensitization or serotonin treatment. Accession numbers are to refseq records for each transcript. Prior results columns summarize previous findings of regulation after long-term sensitization training either early (0-1 hours after training) or late (24 hours after training). ↑ represents up-regulation, “none” represents no regulation, and?? represents time point not tested. Current Results columns presents data from this manuscript for the VC cluster. *Sensorin has not previously been tested in a behavioral context but shows delayed up-regulation after serotonin treatment in cultured sensory neurons [43].

## Materials and Methods

We report how we determined our sample size, all data exclusions (if any), all manipulations, and all measures in these studies [58]. All data for this project is posted to the Open Science Framework (https://osf.io/ts9ea/).

### Sample-Size Determination

Based on effect sizes previously observed [53], [59], [60] in the whole pleural ganglia after long-term sensitization training we set a goal of 10 animals for the 1 hour time point and 12 for the 24-hour time point. These targets provide adequate power (>0.80) for d≥1 and d≥0.9, respectively (based on an uncorrected α = 0.05; within-subjects comparison).

Although 10 animals were collected for the 1-hour time-point, 1 sample did not pass a transcriptional control (see Results), so no tissue from that animal was analyzed further. In addition, 2 samples yielded poor and low-quantity RNA from the VC cluster and the rPl, leaving only n = 7 for these loci at this time-point. At the 24-hour time point, poor and low-quality RNA was obtained from 4 pedal ganglia samples, leaving only n = 8 for this location at this time point.

Although these problems resulted in lower than desired sample sizes, the minimum sample size obtained (n = 7) still provides adequate power (>0.80) for d≥1.3. Moreover, we found that effect sizes in the VC cluster were even larger than those previously observed in the whole pleural ganglia. Power calculations were made using PS Power and Sample Size Calculator 3.0 [61].

### Animals

Animals (75-125g) were obtained from the RSMAS National Resource for *Aplysia* (Miami, FL) and maintained at 16°C in one of two 90-gallon aquariums with continuously circulating artificial sea water (Instant Ocean, Aquarium Systems Inc.). Animals were separately housed in rectangular colanders, fed dried seaweed twice a week, and maintained on a 12 hr light-dark cycle. 2 days prior to any experimental testing, animals were fed to satiation and then food deprived for the remainder of the experiment [62], [63]. To control for batch/shipment effects, animals from at least 2 different shipments were used for each experiment.

### Experimental Manipulations

A one-day long-term sensitization training protocol (Fig. 1C) was conducted similarly to Wainwright et al. [62] but with two differences. First, we stimulated with a 60Hz biphasic square-wave pulse rather than true AC current, as this enabled the use of a constant-current stimulator (WPI High-Current Stimulus Isolation Unit, Model A385, Sarasota, FL) to provide more precise control over stimulus intensity. Second, we used a 90 mA stimulus rather than a 60 mA stimulus. We selected this intensity based on previous work with this stimulator [53], [59] where we observed that a 90 mA stimulus produces more reliable elevations of both behavior and ApC/EBP expression.

Training consisted of 4 rounds of noxious shock applied at 30 minute intervals to one side of the body with a hand-held electrode. Each round of shock consisted of 10 pulses of 500 ms duration at a rate of 1 hz and an amplitude of 90 mA. During the course of each round of shock, the stimulating electrode was slowly moved from anterior (just behind neck) to posterior (just in front of tail) and back to cover nearly the entire surface of that side of the body. Side of training was counterbalanced within each experiment (left- and right-side training equalized for each experiment).

### Behavioral Measurement

As a behavioral outcome, we measured the duration of the tail-elicited siphon-withdrawal reflex (T-SWR) (see [64]). The reflex was evoked by applying a weak shock to one side of the tail using a hand-held stimulator (60 Hz biphasic DC pulse for 500 ms at 2 ma of constant current). T-SWR behavior was measured as the duration of withdrawal from the moment of stimulation to the first sign of siphon relaxation. To characterize changes in T-SWR duration, pre-test and post-test responsiveness was characterized by a series of 8 responses evoked on alternating sides of the body at a 10-min ISI. Scores were split by side of stimulation (trained vs. untrained) and averaged (4 responses/side for each time point characterized).

### Isolation and Processing of Pleural Ganglia RNA

All tissue processing was conducted blind to experimental condition.

CNS samples were harvested 1 hour or 24 hours after training. Briefly, animals were anesthetized with an injection of isotonic MgCl_2_ (50% of body weight), and an incision was then made along the ventral midline to expose the CNS. The pedal/pleural ganglia were then extracted from the left and right side and placed separately in Sylgard coated-dissecting dishes filled with an ice-cold mix of 50% artificial sea water and 50% 333 mM MgCl_2_. Both left and right ganglia were pinned out, and then the sheath overlying the pleural ganglia was resected to allow removal of the VC cluster. The remaining pleural ganglia (rPl) was then removed, and then the pedal ganglion was harvested. For VC and rPl samples, RNA isolation was performed using the RNeasy Plus Micro Kit (Qiagen, Valencia CA) according to manufacturer's instructions. For Pedal samples, tissue was homogenized in Trizol using the Bullet Blender (NextAdvance, Averill Park, NY) and RNA extracted using Direct-Zol Mini RNA Kit (Zymo, Irvine, CA). Because dissection can alter gene expression [52], all tissues were extracted rapidly (<15 minutes per animal), maintained the dissecting dishes on a cold-plate throughout dissection, and dissected both left- and right-sides to the point of extraction before removing ganglia to ensure equal dissection time on both sides. Quantity and quality of RNA was assessed using the NanoDrop 1000 (Thermo Scientific, Wilmington, DE).

### Reverse Transcription-Quantitative PCR

RNA was reverse transcribed using the Maxima First Strand cDNA Synthesis Kit for RT-qPCR with dsDNase (Thermo Scientific, Waltham, MA).

Quantitative PCR was conducted using Maxima SYBR Green/Fluorescein qPCR Master Mix (Thermo Scientific, Waltham, MA)and the MyIQ real time PCR system (Bio-Rad, Los Angeles CA). Primers were validated for correct PCR efficiency and are listed in S1 Table. qPCR samples were analyzed in duplicate and the relative amounts of each transcript were determined using the ddCT method and the Bio-rad IQ5 gene expression analysis (Bio-Rad, Los Angeles CA). All qPCR expression levels were normalized to levels of histone H4, a transcript which is stable during long-term sensitization training [59].

### Statistical Analysis

Behavioral data was analyzed with a 2 (side of training) ×2 (time of test) repeated-measures ANOVA in GraphPad Prism 6.0 (GraphPad Inc., La Jolla, CA) and followed up with Sidak-corrected post-hoc tests.

For analysis of quantitative qPCR data, we analyzed a large number of transcripts at multiple locations within each time point. We thus adopted the same statistical approach we previously used for microarray data [53] to provide strong controls of false positives due to multiple comparisons. First, a fold-change score was calculated for each animal as the ratio of trained to untrained expression. Fold change scores were then log transformed (base 2). This ensures equal weight to both up- and down-regulated measures. Log-fold-change scores were assessed for regulation using an empirical Bayes-moderated *t*-test [65] against a null hypothesis of 0 (when trained and untrained expression are equal, the fold-change score that results is 1, and the log-fold change is 0). Statistical significance was calculated using Benjamini-Hochberg correction for multiple comparisons to maintain a 5% overall false-discovery rate [66]. Confidence intervals for each log-fold change were also calculated and expanded using Benjamini-Hochberg correction for multiple comparisons [67]. These corrections for multiple comparisons were applied with respect to all transcripts tested at all locations for each time point (28 comparisons at 1 hour time point; 20 at the 24 hour time point). Analyses were conducted using limma [68] from the Bioconductor suite of tools [69] for R [70]. Our processing script is posted on the Open Science Framework.

Cohen's *d* is reported as an estimate of effect size, calculated so that positive values indicate increased expression on the trained side.

## Results

### Confirmation of Efficacy of training

We examined transcriptional changes 1 and 24 hours after long-term sensitization training (Fig. 1C). We administered sensitization training to one side of the body from just behind the head to just in front of the tail, a protocol that should activate the majority of VC neurons and produce long-lasting site-specific sensitization over much of the body [16].

**Figure 2:**
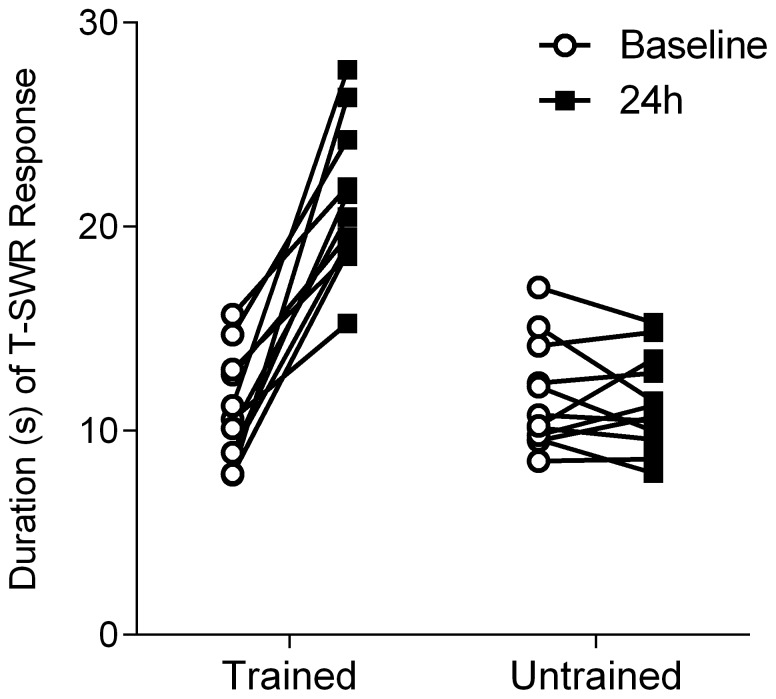
Long-term sensitization training produces a long-lasting but unilateral increase in T-SWR duration. Shown is the T-SWR duration (in seconds) before (baseline, circles) and 24 hours after (squares) long-term sensitization on the trained (left) and untrained sides (*n* = 12 animals). T-SWR durations are similar at baseline for trained and untrained sides. Every animal shows an increase in T-SWR on the trained side following training (positive slopes from baseline to 24 hour measures).

For animals in the 24-hour condition (*n* = 12), we measured the efficacy of training via the tail-elicited siphon-withdrawal response (T-SWR), a test site not included in training and thus representing generalized sensitization. Specifically, T-SWR duration was measured before and 24 hours after training. As expected, training produced a robust increase in T-SWR duration on the side of training but no change in reflex duration on the untrained side (Fig. 2). Specifically, a repeated-measures ANOVA showed the expected interaction between side of training and test phase (*F*(1,11)  = 102.6, *p* = 0.0000007, η^2^ = 26.9). Post-hoc comparisons showed an effect of test phase on the trained side (*t*(11)  = 13.9, *p* = 0.00000003, *d = *2.4) but no change in T-SWR durations on the untrained side (*t*(11)  = 0.33, *p* = 0.94, *d* = −0.1). Thus, consistent with many prior experiments [15], [37], [59], [62], sensitization training produced a robust but unilateral long-term memory.

For animals in the 1 hour condition (*n* = 10), tissue was collected before the efficacy of training could be confirmed behaviorally. Therefore, we adopted ApC/EBP expression in the VC cluster as a transcriptional control. As expected, there was an overall large increase in ApC/EBP expression in the VC cluster on the side of sensitization training (*t*(7) = 3.5, *p* = 0.01, *d* = 1.2, *n* = 8) compared to the control side. In one animal, however, ApC/EBP was not robustly elevated on the trained side. Samples from this animal were discarded and not analyzed further.

### Confirmation of Accuracy of Dissection

To confirm the accuracy of dissection, we compared levels of sensorin mRNA in the VC cluster with the remaining pleural ganglia (rPl) for all animals in the 1 hour condition. Sensorin is selectively expressed in *Aplysia* nociceptors [44], [71]. In the pleural ganglia sensorin is expressed only by VC neurons. Samples were run on the same plate and quantified using the same parameters to ensure comparability of H4-adjusted expression levels.

**Figure 3:**
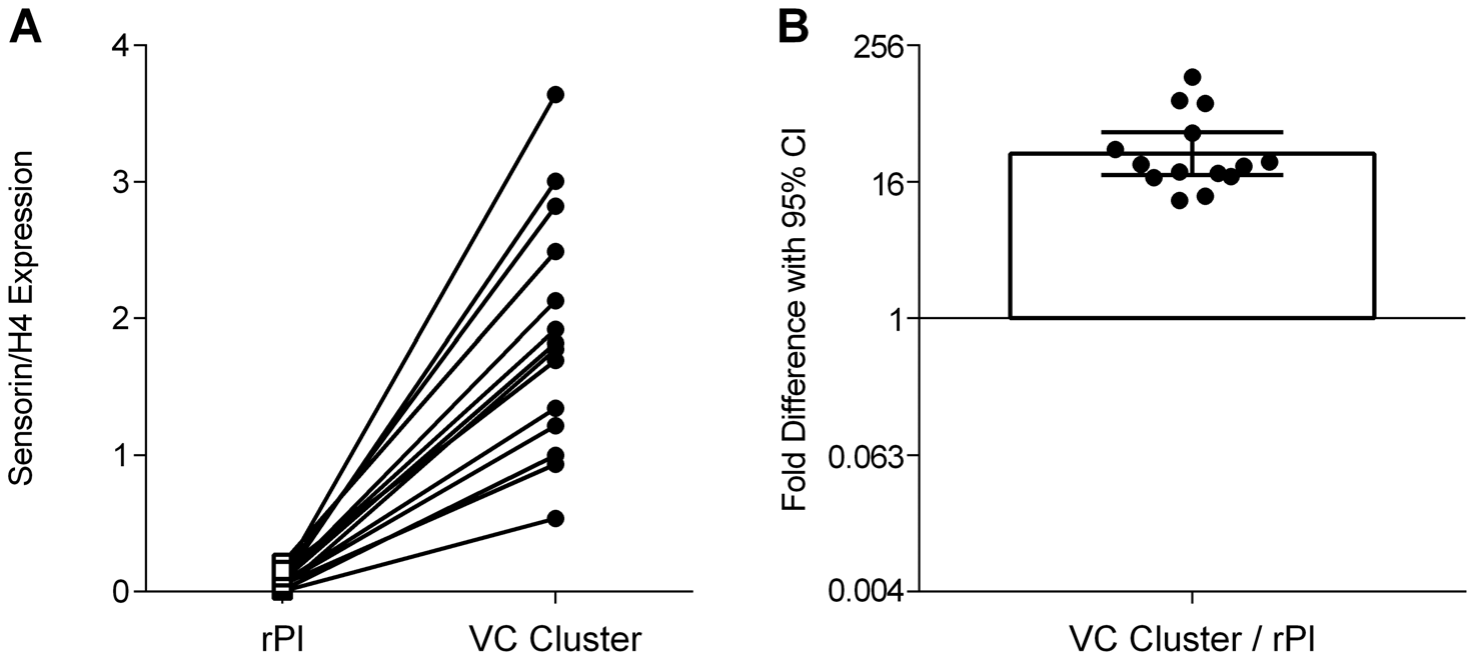
Microdissection of the VC cluster captures most sensorin mRNA in the pleural ganglia. VC clusters were microdissected from the pleural ganglia and expression of sensorin was measured in the VC cluster and the remaining pleural ganglia (rPl). A) Sensorin expression in rPl (squares) and VC cluster (circles) for animals harvested 1 hour after training (*n* = 7, samples from both left and right side of each animal shown). Sensorin expression is normalized to levels of H4, a housekeeping gene unaltered by long-term sensitization training. B) Fold difference in sensorin expression from VC cluster to its corresponding rPl. The bar represents the mean fold difference and the error bar represents the 95% confidence interval of the mean.

As expected, expression of sensorin was abundant in the VC clusters and nearly undetectable in the equivalent rPls (Fig. 3A). This yielded a significant difference when comparing expression across these tissues (*t*(13) = 16.6, *p* = 0.00002, *d* = 4.4). Each rPl had at least an order-of-magnitude less sensorin expression than the VC cluster that had been removed from it (Fig. 3B), with average expression at 3% of what was observed in the VC cluster. This data indicates that dissection reliably captured almost the entire VC cluster, and that the rPl samples do not reflect a large contribution from accidentally omitted VC neurons.

### Transcriptional changes 1 hour after long-term sensitization training

We first examined transcriptional changes occurring 1 hour after long-term sensitization training, surveying the complete set of 10 transcripts previously found to be rapidly regulated by training in either VC neurons or the pleural ganglion as a whole (see Table 1).

**Figure 4:**
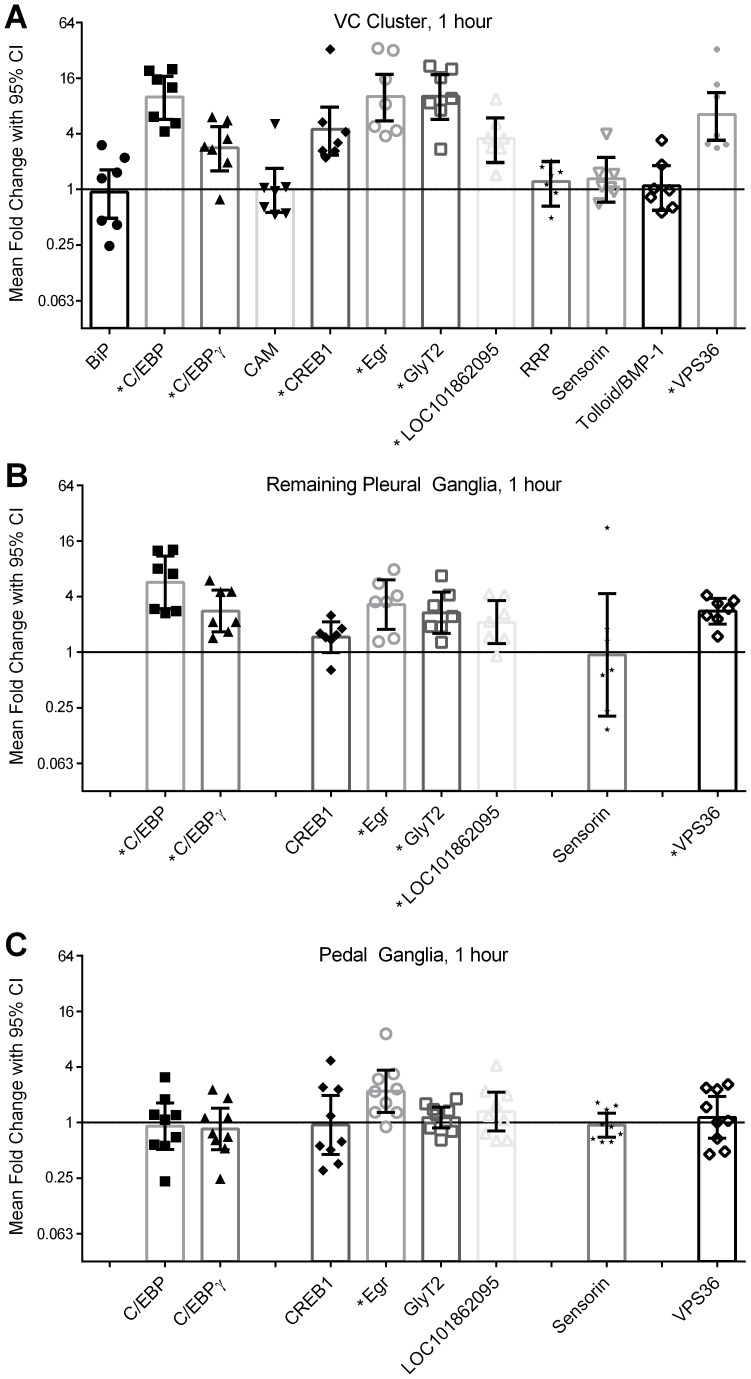
Changes in transcription 1 hour after long-term sensitization training. Shown is raw data, means, and adjusted 95% confidence intervals for each transcript measured 1 hour after long-term sensitization training. Samples were harvested from 3 CNS loci: A) the VC cluster (*n* = 7), B) the remaining pleural ganglia (*n* = 7), and C) the pedal ganglia (*n* = 9). Only transcripts significantly up-regulated in the VC cluster were tested in the pleural ganglia and pedal ganglia, producing some empty columns in Panels B and C. * indicates significant regulation at *p* <0.05 with Benjamini-Hochberg correction for multiple comparisons for all measurements presented in this figure. Confidence intervals are also expanded for multiple comparisons to maintain an overall false-discovery rate of 0.05.

As expected (Fig. 4A), in the VC cluster there was strong and consistent up-regulation of ApC/EBP (*p* = 0.000000007, *d* = 2.9), ApC/EBPγ (*p* = 0.001, *d* = 1.3), ApCREB1 (*p* = 0.00004, *d* = 1.8), ApEgr (*p* = 0.00000002, *d* = 2.7), ApGlyT2 (*p* = 0.000000007, *d* = 2.9), LOC101862095 (*p* = 0.0001, *d* = .6), and ApVPS36 (*p* = 0.000001, *d* = 2.2). Unexpectedly, we did not observe regulation of ApCaM (*p* = 0.99, *d* = 0.0), ApRRP (*p* = 0.67, *d* = 0.3), or ApTBL-1 (*p* = 0.87, *d* = 0.1) at this time point. Each of these transcripts has been previously reported to be up-regulated immediately (0 hours) in VC neurons after similar long-term sensitization training [18], [56]. As negative controls, we tested ApBiP and sensorin, which show delayed increases in transcription following behavioral testing and serotonin exposure, respectively. As expected, neither of these transcripts were strongly regulated in the VC cluster at 1 hour after training (ApBip: *p* = 0.88, *d* = −0.1; sensorin: *p* = 0.50, *d* = 0.3).

We next examined transcripts regulated in the VC cluster for similar regulation in the remaining pleural ganglion (Fig. 4B). We found that results in the rPl largely mirrored what we observed in the VC cluster: significant up-regulation of ApC/EBP (*p* = 0.000001, *d* = 2.2), ApC/EBPγ, (*p* = 0.001, *d* = 1.3), ApEgr (*p* = 0.0003, *d* = 1.5), ApGlyT2 (*p* = 0.001, *d* = 1.3), LOC101862095 (*p* = 0.01, *d* = 1.0), and ApVP36 (*p* = .0008, *d* = 1.4). The one possible exception was ApCREB, which was up-regulated in 6 of 7 samples, but did not reach statistical significance (*p* = 0.26, *d* = 0.5), possibly due to low power. Effect sizes observed in the rPl were consistently smaller than what was observed in the VC cluster. Although rPl samples showed similar transcriptional up-regulation as observed in VC samples, expression was not consistently correlated across VC and rPl samples (*r* = −0.21, 0.28, 0.19, 0.43, −0.13, 0.61, and −0.51 for ApC/EBP, ApC/EBPγ, ApCREB1, ApEgr, ApGlyT2, LOC101862095, and VPS36, respectively). Fold-change scores in sensorin in the rPL are also plotted in Fig. 4B, and did not reach statistical significance (*p* = 0.89, *d* = −0.04). Note, however, that the very low expression of sensorin in the rPL produced wide variation in fold-change scores and thus makes this result difficult to interpret.

In the pedal ganglion (Fig. 4C), we found limited transcriptional regulation at this time point. Only ApEgr showed strong changes due to training (*p* = 0.004, *d* = 1.1). All other transcripts which had shown robust regulation in the VC cluster were not very strongly regulated in the pedal ganglia (*p*>0.42 for each comparison, *d* from −0.2 to 0.4). We were also able to detect sensorin expression in the pedal ganglion; this is due to strong expression in the VC dendrites located in the pedal ganglia [44]. We did not observe, however, a strong influence of sensitization training on sensorin in the pedal ganglia (*p* = 0.88, *d* = −0.2).

### Transcriptional changes 24 hours after long-term sensitization training

**Figure 5:**
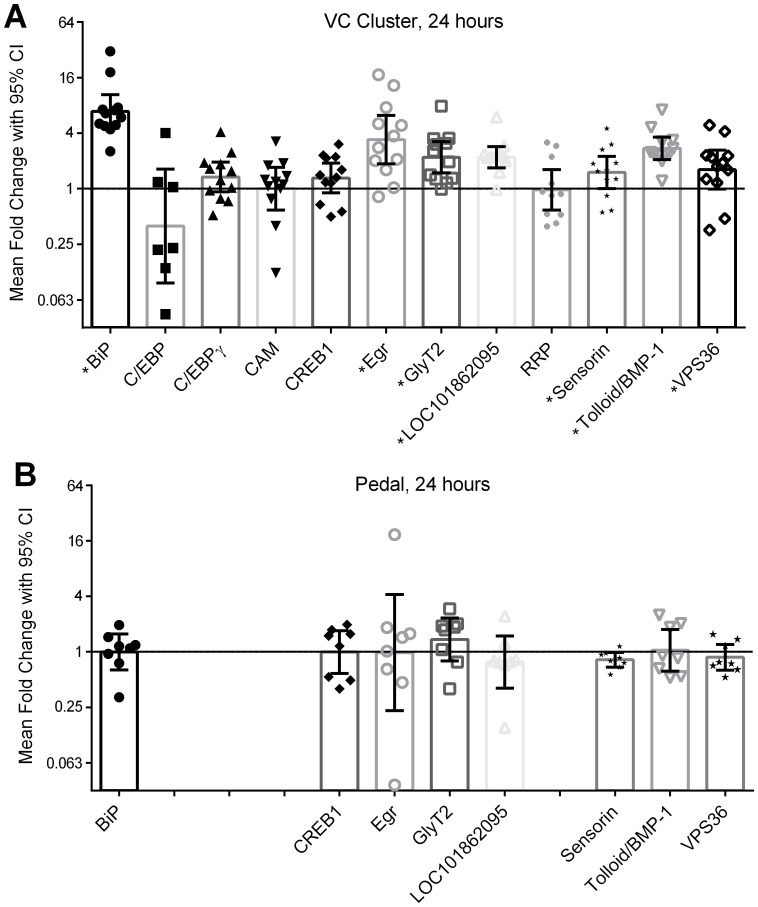
Changes in transcription 24 hours after long-term sensitization training. Shown is raw data, means, and adjusted 95% confidence intervals for each transcript measured 24 hours after long-term sensitization training. Samples were harvested from 2 CNS loci: A) the VC cluster (*n* = 12) and B) the pedal ganglia (*n* = 8). Only transcripts significantly up-regulated in the VC cluster were tested in the pedal ganglia, producing some empty columns in panel B. ApC/EBP was undetectable in the VC cluster of 5 samples, so only the remaining 7 samples are presented here. * indicates significant regulation at *p* <0.05 with Benjamini-Hochberg correction for multiple comparisons for all measurements presented in this figure. Confidence intervals are also expanded for multiple comparisons to maintain an overall false-discovery rate of 0.05.

We next examined the persistent transcriptional changes occurring 24 hours after long-term sensitization training. In the VC cluster (Fig. 5A), late-response transcripts ApBiP (*p* = 0.000000008, *d* = 5.1) and sensorin (*p* = 0.047, *d* = 1.1) were up-regulated. In addition, the known early-response transcript ApC/EBP was completely undetectable in 5 (of 12) samples. Note the variation in the 7 remaining ApC/EBP measures is highly unstable due to low expression and so must be interpreted with caution.

Strong long-term regulation in the VC cluster was evident for ApEgr (*p* = 0.00009, *d = *2.6), ApGlyT2 (*p* = 0.005, *d* = 1.3), LOC101862095 (*p* = 0.0001, *d* = 1.9), ApTBL-1 (*p = *0.000006, *d* = 2.3) and VPS36 (*p* = 0.04, *d* = 0.62). The other transcripts tested did not show strong regulation in the VC cluster at this time point: ApC/EBPγ (*p = *0.12, *d = *0.5), APCaM (*p* = 0.99, *d = *0.0), ApCREB1 (*p = *0.15, *d = *0.46), and ApRRP (*p = *0.90, *d = *0.0).

All transcripts regulated in the VC cluster were also tested in the pedal ganglia (Fig. 5B), but no strong regulation was observed: ApBiP (*p = *0.99, *d = *0.0), ApEgr (*p* = 0.97, *d = *0.0), ApGlyT2 (*p = *0.21, *d = *0.5), LOC101862095 (*p = *0.34, *d* = −0.4), ApTBL-1 (*p = *0.87, *d = *0.1), VPS36 (*p* = 0.45, *d* = −0.3). Sensorin expression was reduced on the trained side in 8 of 9 animals tested, but this was not significant after correction for multiple comparisons (*p = *0.34, *d* = −0.9). ApCREB1 was also tested, but also did not show strong regulation in the pedal ganglia (*p* = 0.98, *d = *0.0).

rPl samples were not tested at this time point for a number of reasons: 1) because the results at 1 hour had so closely mirrored those found in the VC cluster, 2) because there is RNA export from the VC cell bodies following treatments which mimic sensitization [57], which could make these samples less independent of the VC cluster, and 3) the abundance of RNA harvested from these rPl samples was low.

## Discussion

We used qPCR to begin to dissect how the transcriptional response to long-term sensitization training is distributed across CNS locations relevant to defensive withdrawal. The VC cluster of nociceptors shows the strongest and most diverse transcriptional response to training. This makes sense given the variety of physiological changes evoked in the VCs following sensitization training [19], [36], [37], [72]. It seems clear, though, that the VCs are not the only site of transcriptional regulation. At one hour after training we observed strong up-regulation of a number of transcripts in the remaining pleural ganglia as well as a transient increase in ApEgr expression in the pedal ganglia. Although both of these sites contain processes of the VC nociceptors, it seems unlikely that this could account for the regulation observed. First, almost no sensorin was detectable in the rPl samples, suggesting minimal contribution from the VCs. Second, expression changes in the rPl were not consistently correlated with those observed in the VCs. Finally, in cultured VC neurons it takes more than two hours for transcripts induced by serotonin treatment to be exported from the cell bodies to the neurites at a detectable level [57]. Taken together, it seems likely that the rapid transcriptional changes we observed in the rPl and pedal samples are not driven by the VC nociceptors.

What cell types could account for the regulation observed in the rPl and Pedal ganglia? One possibility is that these changes reflect additional components of defensive reflex components: interneurons within the rPl, and motor neurons and serotonergic modulatory neurons (ref 34,) within the pedal ganglia. This is especially intriguing given that transcripts persistently regulated in the VC cluster do *not* seem to be strongly regulated in the pedal ganglion. This could indicate that a common initial transcriptional response leads to distinctive endpoints across defensive circuits, perhaps mediating the differential physiological changes [36] that occur after long-term sensitization training. It must also be kept in mind, however, that both the rPl and the pedal ganglia contain many neurons not directly related to defensive withdrawal reflexes. Thus, the transcriptional changes we observed in these samples could represent processes occurring outside of the T-SWR circuit, perhaps related to some of the additional behavioral effects[21], [22], [73] of long-term sensitization training. We are planning on using both microarray and single-cell PCR to better elucidate the different transcriptional responses in different neural subtypes that occur after long-term sensitization training.

Our results within the VC cluster help extend our knowledge of several transcriptional changes induced by long-term sensitization training. This is the first evidence that ApC/EBPγ, ApCREB1, ApEgr, ApGlyT2, LOC101862095 and VPS36 are specifically regulated in the VC nociceptors (previous work was on whole pleural ganglia only). In addition, this work is the first to examine long-term regulation of ApC/EBPγ, ApCaM, ApGlyT2, LOC101862095, ApRRP, and VPS36. Are these transcriptional changes in the VC cluster related to site-specific sensitization induced by the combination of serotonergic modulation as well as training-induced VC activity? Or are these transcriptional changes related to generalized sensitization that seems largely due to serotonergic modulation alone? The training stimulus was applied to nearly the entire surface of one side of the body, so the majority of the VCs collected should have been activated by the training stimulus. Thus, the transcriptional changes we observed may be primarily driven by site-specific sensitization. It is not possible, however, to definitively attribute these changes to site-specific or generalized sensitization without further partitioning VC samples during dissection. While this would be an interesting line of inquiry, it is important to note that the both forms of memories seems to have similar physiological and molecular substrates.

The temporal patterns of regulation observed in the VC cluster encompass all three possible patterns given the time points surveyed: 1) rapid but transient (ApC/EBP, ApC/EBPγ, and ApCREB1), 2) delayed but persistent (ApBiP, sensorin, and ApTBL-1), and 3) both rapid *and* persistent (ApEgr, ApGlyT2, LOC101862095, and VPS36). What is the basis for these different temporal profiles of regulation? One possibility is that different upstream promoters confer sensitivity to transcription factors that are expressed either early or late after sensitization training. We are currently using bioinformatics and promoter analysis to identify specific transcription factor and promoter motifs that could specify these three different temporal profiles of regulation. The rapid and persistent pattern is particularly intriguing, as this could represent transcripts important for sustaining the transcriptional trace of long-term sensitization memory.

In contrast to prior findings [18], [56], we did not observe a rapid increase in the expression of ApCaM, ApRRP, or ApTBL-1 after long-term sensitization training. This could be due to a difference in time-points selected, as the original findings harvested VC neurons immediately after training whereas we harvested 1 hour after training. It seems somewhat unlikely, though, that regulation of these transcripts could be so transient. Another possibility is that our sample size was inadequate to reliably detect regulation of these transcripts. Indeed, the mean fold change previously reported for these changes are modest (33%, 41%, and 29%, respectively). However, the effect sizes observed (mean fold change relative to variation in expression) were large (*d* = 1.7, 1.7, and 1.4, respectively), and within the range for which our sample size provides reasonable statistical power. We believe the most likely explanation is that subtle differences in training could have led to a slightly delayed development of regulation in these transcripts, too late to be captured at our 1 hour time point.

Although we did not observe a rapid regulation of ApTBL-1, we did observe a strong increase in expression at 24 hours after training. This is in contrast to what has been previously observed with serotonin treatment in VC nociceptors, where ApTBL-1 expression is rapidly induced but falls to baseline within 3 hours after treatment [56]. This difference is notable, because it suggests that in a behavioral context ApTBL-1 could produce sustained activation of TGF-β1. This is important because only sustained TGF- β 1 exposure (24 hours but not 4 hours), produces the same long-term facilitation of sensory-to-motor synapses [74] as long-term sensitization training.

Another surprising finding is a lack of sustained up-regulation of ApCREB1. ApCREB1 has previously been proposed to play a role not only in the induction of sensitization memory but also in consolidation and maintenance through a transcriptional positive-feedback loop: ApCREB1 can bind to its own promoter to increase its own expression [75], is persistently up-regulated by serotonin treatments that produce long-term facilitation [75], [76], and knockdown of ApCREB1 mRNA 10 hours after treatment abolishes long-term facilitation[76]. Nevertheless, we previously failed to observe persistent up-regulation of ApCREB1 24 h after long-term sensitization training in the pleural ganglia [59]. Our results here extend this finding, with no strong regulation of ApCREB1 observable 24 hours after training in either the VC nociceptors or pedal ganglion. It is possible that regulation of ApCREB1 is too subtle to detect after behavioral training with the sample sizes we used. However, it is notable that ApC/EBP, a transcript activated by ApCREB1 in partnership with CREB-Binding Protein [77], also shows only a transient increase in expression [59], suggesting that a different transcriptional signal may be responsible for maintaining long-term sensitization memory.

Microdissecting the VC cluster for separate analysis has been previously used to ensure transcriptional changes are specific to the T-SWR circuit [18], [51], [56]. Our data show that despite the possibility of variation in dissection quality, isolating the VC cluster greatly increases the signal-to-noise for transcriptional analysis. The effect sizes within the VC cluster were always larger than what has been previously reported across the entire pleural ganglia. For example, we have previously examined ApEgr regulation at 1 and 24 hours after training in the pleural ganglia and obtained effect sizes of 1.6 and 0.7 [53], [55], respectively. Within the VC cluster, however, we observed effect sizes of 2.5 and 1.3, respectively. This data suggests that microdissection of the VC cluster, though time-consuming and frequently leading to low yields of RNA, can dramatically increase experimental power for studying the transcriptional effects of sensitization training.

## Supporting Information

S1 Table **List of transcripts and qPCR primers.**
(XLSX)
